# Benzyl­aminium perchlorate–18-crown-6 (1/1)

**DOI:** 10.1107/S1600536811007082

**Published:** 2011-03-02

**Authors:** Wen-zhe Wang, Xiu-juan Li, Na Wang

**Affiliations:** a RiZhao Polytechnic, RiZhao 276826, People’s Republic of China

## Abstract

In the title compound, C_7_H_10_N^+^·ClO_4_^−^·C_20_H_24_O_6_, the proton­ated benzyl­amine cation forms a rotator–stator complex with the 18-crown-6 (1,4,7,10,13,16-hexa­oxacyclo­octa­deca­ne) mol­ecule *via* N—H⋯O hydrogen bonds. The cations are associated *via* weak C—H⋯π inter­actions, forming chains parallel to [011], while the perclorate anions are located between these chains.

## Related literature

For a related structure, see: Ge *et al.*. (2010 ▶).
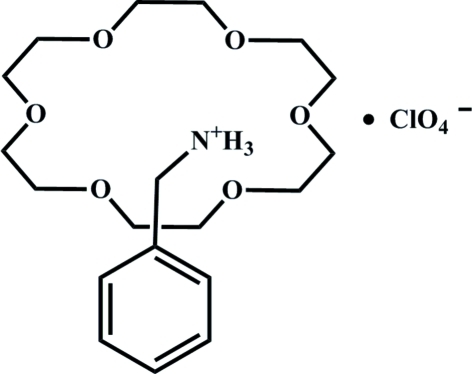


## Experimental

### 

#### Crystal data

C_7_H_10_N^+^·ClO_4_^−^·C_12_H_24_O_6_
*M* *_r_* = 471.92
Triclinic, 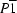

*a* = 9.3482 (19) Å
*b* = 10.948 (2) Å
*c* = 12.071 (2) Å
α = 76.71 (3)°
β = 86.64 (3)°
γ = 78.27 (3)°
*V* = 1177.1 (4) Å^3^
*Z* = 2
Mo *K*α radiation
μ = 0.21 mm^−1^
*T* = 298 K
0.40 × 0.30 × 0.20 mm

#### Data collection

Rigaku SCXmini diffractometer
Absorption correction: multi-scan (*CrystalClear*; Rigaku, 2005 ▶) *T*_min_ = 0.926, *T*_max_ = 0.958
12262 measured reflections
5391 independent reflections
3637 reflections with *I* > 2σ(*I*)
*R*_int_ = 0.036

#### Refinement

*R*[*F* ^2^ > 2σ(*F* ^2^)] = 0.058
*wR*(*F* ^2^) = 0.157
*S* = 1.03
5391 reflections
292 parameters
3 restraints
H atoms treated by a mixture of independent and constrained refinement
Δρ_max_ = 0.49 e Å^−3^
Δρ_min_ = −0.32 e Å^−3^

### 

Data collection: *CrystalClear* (Rigaku, 2005 ▶); cell refinement: *CrystalClear*; data reduction: *CrystalClear*; program(s) used to solve structure: *SHELXS97* (Sheldrick, 2008 ▶); program(s) used to refine structure: *SHELXL97* (Sheldrick, 2008 ▶); molecular graphics: *SHELXTL* (Sheldrick, 2008 ▶); software used to prepare material for publication: *SHELXTL*.

## Supplementary Material

Crystal structure: contains datablocks I, global. DOI: 10.1107/S1600536811007082/kp2306sup1.cif

Structure factors: contains datablocks I. DOI: 10.1107/S1600536811007082/kp2306Isup2.hkl

Additional supplementary materials:  crystallographic information; 3D view; checkCIF report

## Figures and Tables

**Table 1:** Hydrogen-bond geometry (Å, °) *Cg*1 is the centroid of the C14–C19 benzene ring.

*D*—H⋯*A*	*D*—H	H⋯*A*	*D*⋯*A*	*D*—H⋯*A*
N1—H1⋯O4	0.86 (2)	2.18 (2)	3.004 (3)	161 (3)
N1—H2⋯O2	0.84 (2)	2.11 (2)	2.938 (3)	174 (3)
N1—H3⋯O1	0.85 (2)	2.47 (2)	2.955 (3)	117 (2)
N1—H3⋯O6	0.85 (2)	2.07 (2)	2.885 (3)	162 (3)
C13—H13*B*⋯*Cg*1^i^	0.97	2.99	3.545 (3)	117

Symmetry code: (i) 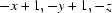.

**Fig. 1.**
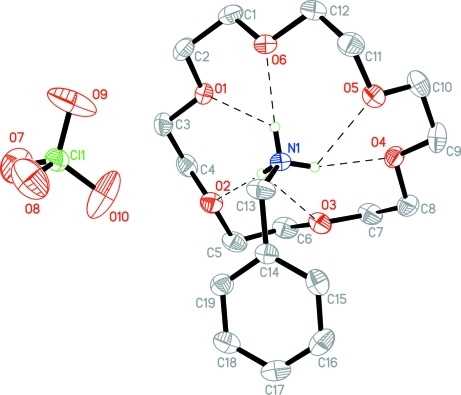
The molecular structure of the title compound with the atomic numbering scheme. Displacement ellipsoids are drawn at the 30% probability level. N-bound H atoms are shown as small spheres of arbitrary radii, all other H atoms have been omitted for clarity. Dashed lines indicate N—H···O hydrogen bonds.

**Fig. 2.**
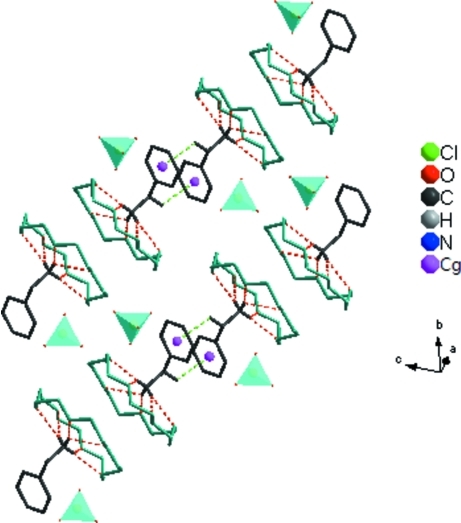
A view of crystal packing with stacking parallel to [0 1 1]. Dashed lines indicate hydrogen bonds and C—H···π interactions. Cg denotes the ring centroid. [symmetry code: (i) -x + 1, -y + 1, -z]

## supplementary crystallographic information

### Comment

The asymmetric unit of the title compound, (I), contains a 1:1 (C_7_H_10_N^+^)(18-crown-6) adduct forming the supramolecular cation and a perchlorate anion (Fig. 1). The 18-crown-6 molecule possesses a boat conformation, while the ammonium atom is in a forward perching position. The supramolecular cation is involved in three bifurcated hydrogen bonds, each to two adjacent oxygen atoms of the crown ring, with the hydrogen bond length from 2.885 (3) Å- 3.004 (3)Å (Table 1).

The pairwise face-to-face supramolecular cations are linked by C—H···*Cg*^i^ [symmetry code: (i) -*x* + 1, -*y* + 1, -*z*] interactions with C···centroid distances of 3.545 (3) Å, forming one-dimensional chains parallel to [0 1 1] (Fig. 2). The perchlorate anions are located between the chains of cations.

### Experimental

Benzylamine (1.07 g, 10 mmol) was firstly dissolved in methanol (20 mL), to which perchloraic acid aqueous solution was dropped slowly with stirring until pH of the solution gradually changed to *ca* 7.18-crown-6 (2.64 g 10 mmol) methanol solution was mixed. Methanol was added until the precipitated substrate disappeared, then the solution was allowed to slowly evaporate at room temperature until prisms of the title compound were grown.

### Refinement

Positional parameters of all the H atoms for C atoms were calculated geometrically and were allowed to ride on the C atoms to which they are bonded, with *U*_iso_(H) = 1.2Ueq(C). All ammonium H atoms were found in a difference Fourier map and refined with restraints for the N—H distances of 0.87 (2) Å.

### Crystal data

**Table:**

C_7_H_10_N^+^·ClO_4_^−^·C_12_H_24_O_6_	*Z* = 2
*M**_r_* = 471.92	*F*(000) = 504
Triclinic, *P*1	*D*_x_ = 1.332 Mg m^−^^3^
Hall symbol: -P 1	Mo *K*α radiation, λ = 0.71073 Å
*a* = 9.3482 (19) Å	Cell parameters from 5069.5 reflections
*b* = 10.948 (2) Å	θ = 3.2–27.6°
*c* = 12.071 (2) Å	µ = 0.21 mm^−^^1^
α = 76.71 (3)°	*T* = 298 K
β = 86.64 (3)°	Prism, colourless
γ = 78.27 (3)°	0.40 × 0.30 × 0.20 mm
*V* = 1177.1 (4) Å^3^	

### Data collection

**Table:**

Rigaku SCXmini diffractometer	5391 independent reflections
Radiation source: fine-focus sealed tube	3637 reflections with *I* > 2σ(*I*)
graphite	*R*_int_ = 0.036
Detector resolution: 13.6612 pixels mm^-1^	θ_max_ = 27.5°, θ_min_ = 3.2°
ω scans	*h* = −12→12
Absorption correction: multi-scan (*CrystalClear*; Rigaku, 2005)	*k* = −14→14
*T*_min_ = 0.926, *T*_max_ = 0.958	*l* = −15→15
12262 measured reflections	

### Refinement

**Table:**

Refinement on *F*^2^	Primary atom site location: structure-invariant direct methods
Least-squares matrix: full	Secondary atom site location: difference Fourier map
*R*[*F*^2^ > 2σ(*F*^2^)] = 0.058	Hydrogen site location: inferred from neighbouring sites
*wR*(*F*^2^) = 0.157	H atoms treated by a mixture of independent and constrained refinement
*S* = 1.03	*w* = 1/[σ^2^(*F*_o_^2^) + (0.0636*P*)^2^ + 0.4966*P*] where *P* = (*F*_o_^2^ + 2*F*_c_^2^)/3
5391 reflections	(Δ/σ)_max_ < 0.001
292 parameters	Δρ_max_ = 0.49 e Å^−^^3^
3 restraints	Δρ_min_ = −0.32 e Å^−^^3^

### Special details

**Table:**

Geometry. All e.s.d.'s (except the e.s.d. in the dihedral angle between two l.s. planes) are estimated using the full covariance matrix. The cell e.s.d.'s are taken into account individually in the estimation of e.s.d.'s in distances, angles and torsion angles; correlations between e.s.d.'s in cell parameters are only used when they are defined by crystal symmetry. An approximate (isotropic) treatment of cell e.s.d.'s is used for estimating e.s.d.'s involving l.s. planes.
Refinement. Refinement of *F*^2^ against ALL reflections. The weighted *R*-factor *wR* and goodness of fit *S* are based on *F*^2^, conventional *R*-factors *R* are based on *F*, with *F* set to zero for negative *F*^2^. The threshold expression of *F*^2^ > σ(*F*^2^) is used only for calculating *R*-factors(gt) *etc*. and is not relevant to the choice of reflections for refinement. *R*-factors based on *F*^2^ are statistically about twice as large as those based on *F*, and *R*- factors based on ALL data will be even larger.

### Fractional atomic coordinates and isotropic or equivalent isotropic displacement parameters (Å^2^)

**Table:**

	*x*	*y*	*z*	*U*_iso_*/*U*_eq_	
Cl1	0.09437 (7)	0.73666 (6)	0.23044 (6)	0.0524 (2)	
N1	0.4131 (2)	0.2586 (2)	0.25044 (18)	0.0396 (4)	
H3	0.328 (2)	0.241 (2)	0.262 (2)	0.055 (8)*	
H2	0.434 (3)	0.280 (3)	0.309 (2)	0.080 (11)*	
H1	0.473 (3)	0.191 (2)	0.242 (2)	0.061 (8)*	
C1	0.0242 (3)	0.2263 (3)	0.3324 (2)	0.0539 (6)	
H1A	0.0421	0.1450	0.3874	0.065*	
H1B	−0.0765	0.2435	0.3083	0.065*	
C2	0.0486 (3)	0.3295 (3)	0.3861 (2)	0.0570 (7)	
H2A	0.0375	0.4099	0.3300	0.068*	
H2B	−0.0228	0.3398	0.4465	0.068*	
C3	0.2209 (3)	0.3866 (3)	0.4913 (2)	0.0574 (7)	
H3A	0.1504	0.3930	0.5530	0.069*	
H3B	0.2119	0.4703	0.4403	0.069*	
C4	0.3716 (3)	0.3441 (3)	0.5380 (2)	0.0563 (7)	
H4A	0.3864	0.3972	0.5889	0.068*	
H4B	0.3844	0.2562	0.5811	0.068*	
C5	0.6237 (3)	0.3138 (3)	0.4840 (3)	0.0619 (7)	
H5A	0.6322	0.3391	0.5549	0.074*	
H5B	0.6854	0.3574	0.4276	0.074*	
C6	0.6772 (3)	0.1724 (3)	0.5013 (2)	0.0618 (7)	
H6A	0.7751	0.1489	0.5319	0.074*	
H6B	0.6138	0.1273	0.5549	0.074*	
C7	0.7380 (3)	0.0069 (3)	0.4014 (3)	0.0622 (8)	
H7A	0.6859	−0.0457	0.4588	0.075*	
H7B	0.8397	−0.0112	0.4232	0.075*	
C8	0.7269 (3)	−0.0241 (3)	0.2899 (3)	0.0605 (7)	
H8A	0.7716	0.0334	0.2311	0.073*	
H8B	0.7778	−0.1111	0.2916	0.073*	
C9	0.5564 (3)	−0.0628 (3)	0.1715 (2)	0.0621 (7)	
H9A	0.5974	−0.1538	0.1880	0.075*	
H9B	0.6062	−0.0219	0.1048	0.075*	
C10	0.3972 (3)	−0.0412 (3)	0.1491 (2)	0.0585 (7)	
H10A	0.3805	−0.0887	0.0941	0.070*	
H10B	0.3450	−0.0702	0.2189	0.070*	
C11	0.1953 (3)	0.1244 (3)	0.0785 (2)	0.0574 (7)	
H11A	0.1734	0.0645	0.0370	0.069*	
H11B	0.1754	0.2091	0.0287	0.069*	
C12	0.0963 (3)	0.1234 (3)	0.1806 (2)	0.0574 (7)	
H12A	−0.0047	0.1405	0.1574	0.069*	
H12B	0.1167	0.0400	0.2323	0.069*	
C13	0.4150 (3)	0.3677 (3)	0.1516 (2)	0.0558 (7)	
H13A	0.3409	0.4401	0.1630	0.067*	
H13B	0.3909	0.3441	0.0833	0.067*	
C14	0.5614 (2)	0.4067 (2)	0.13535 (19)	0.0410 (5)	
C15	0.6709 (3)	0.3499 (2)	0.0712 (2)	0.0534 (6)	
H15A	0.6544	0.2862	0.0369	0.064*	
C16	0.8057 (3)	0.3872 (3)	0.0573 (2)	0.0619 (7)	
H16A	0.8787	0.3488	0.0135	0.074*	
C17	0.8306 (3)	0.4798 (3)	0.1078 (2)	0.0595 (7)	
H17A	0.9213	0.5035	0.0996	0.071*	
C18	0.7231 (3)	0.5378 (3)	0.1702 (2)	0.0590 (7)	
H18A	0.7402	0.6018	0.2038	0.071*	
C19	0.5883 (3)	0.5018 (2)	0.1837 (2)	0.0503 (6)	
H19A	0.5152	0.5424	0.2260	0.060*	
O1	0.19248 (17)	0.29632 (15)	0.43136 (15)	0.0486 (4)	
O2	0.47524 (19)	0.35379 (16)	0.44750 (14)	0.0512 (4)	
O3	0.67755 (19)	0.13841 (15)	0.39507 (15)	0.0521 (4)	
O4	0.57664 (17)	−0.01084 (16)	0.26576 (14)	0.0482 (4)	
O5	0.34709 (18)	0.09153 (16)	0.10618 (14)	0.0493 (4)	
O6	0.11943 (17)	0.21897 (16)	0.23658 (15)	0.0490 (4)	
O7	0.0730 (3)	0.7953 (3)	0.3246 (2)	0.1013 (8)	
O8	0.0789 (4)	0.8327 (2)	0.1295 (2)	0.1123 (10)	
O9	−0.0107 (4)	0.6649 (4)	0.2327 (3)	0.1528 (15)	
O10	0.2345 (3)	0.6655 (4)	0.2346 (3)	0.1501 (15)	

### Atomic displacement parameters (Å^2^)

**Table:**

	*U*^11^	*U*^22^	*U*^33^	*U*^12^	*U*^13^	*U*^23^
Cl1	0.0508 (4)	0.0502 (4)	0.0589 (4)	−0.0132 (3)	−0.0051 (3)	−0.0139 (3)
N1	0.0340 (11)	0.0437 (11)	0.0426 (11)	−0.0117 (9)	0.0003 (9)	−0.0089 (9)
C1	0.0352 (12)	0.0615 (16)	0.0613 (16)	−0.0114 (11)	0.0027 (11)	−0.0054 (13)
C2	0.0385 (13)	0.0610 (16)	0.0668 (17)	0.0017 (12)	0.0068 (12)	−0.0165 (14)
C3	0.0624 (17)	0.0521 (15)	0.0619 (17)	−0.0090 (13)	0.0110 (13)	−0.0263 (13)
C4	0.0761 (19)	0.0547 (15)	0.0440 (14)	−0.0201 (14)	0.0026 (13)	−0.0176 (12)
C5	0.0598 (17)	0.0690 (18)	0.0655 (18)	−0.0257 (14)	−0.0162 (14)	−0.0173 (14)
C6	0.0586 (16)	0.0673 (18)	0.0581 (17)	−0.0166 (14)	−0.0223 (13)	−0.0017 (14)
C7	0.0482 (15)	0.0481 (15)	0.085 (2)	−0.0051 (12)	−0.0271 (14)	−0.0004 (14)
C8	0.0390 (14)	0.0507 (15)	0.088 (2)	0.0001 (11)	−0.0036 (13)	−0.0139 (14)
C9	0.0764 (19)	0.0549 (16)	0.0515 (16)	0.0071 (14)	0.0016 (14)	−0.0227 (13)
C10	0.0798 (19)	0.0498 (15)	0.0509 (15)	−0.0147 (13)	−0.0110 (13)	−0.0171 (12)
C11	0.0550 (16)	0.0655 (17)	0.0556 (16)	−0.0152 (13)	−0.0123 (13)	−0.0153 (13)
C12	0.0465 (14)	0.0617 (16)	0.0717 (18)	−0.0219 (12)	−0.0040 (13)	−0.0195 (14)
C13	0.0471 (14)	0.0520 (15)	0.0625 (17)	−0.0177 (12)	−0.0168 (12)	0.0101 (12)
C14	0.0422 (12)	0.0407 (12)	0.0368 (12)	−0.0106 (10)	−0.0073 (9)	0.0019 (10)
C15	0.0675 (17)	0.0455 (14)	0.0469 (14)	−0.0100 (12)	−0.0067 (12)	−0.0092 (11)
C16	0.0521 (16)	0.0711 (19)	0.0523 (16)	0.0009 (14)	0.0109 (12)	−0.0070 (14)
C17	0.0477 (15)	0.0762 (19)	0.0526 (16)	−0.0236 (14)	0.0010 (12)	−0.0006 (14)
C18	0.0638 (17)	0.0609 (17)	0.0587 (16)	−0.0287 (14)	−0.0003 (14)	−0.0118 (14)
C19	0.0492 (14)	0.0542 (15)	0.0483 (14)	−0.0125 (12)	0.0054 (11)	−0.0122 (12)
O1	0.0421 (9)	0.0446 (9)	0.0602 (11)	−0.0030 (7)	0.0027 (8)	−0.0194 (8)
O2	0.0553 (10)	0.0568 (10)	0.0448 (10)	−0.0166 (8)	−0.0035 (8)	−0.0122 (8)
O3	0.0542 (10)	0.0435 (9)	0.0558 (11)	−0.0100 (8)	−0.0175 (8)	−0.0010 (8)
O4	0.0393 (9)	0.0531 (10)	0.0522 (10)	−0.0018 (7)	−0.0003 (7)	−0.0184 (8)
O5	0.0518 (10)	0.0485 (10)	0.0507 (10)	−0.0130 (8)	−0.0025 (8)	−0.0138 (8)
O6	0.0388 (9)	0.0510 (10)	0.0611 (11)	−0.0157 (7)	0.0037 (8)	−0.0153 (8)
O7	0.0982 (18)	0.133 (2)	0.0844 (17)	−0.0079 (16)	−0.0136 (14)	−0.0580 (17)
O8	0.166 (3)	0.0825 (17)	0.0832 (18)	−0.0337 (18)	−0.0295 (18)	0.0073 (14)
O9	0.179 (3)	0.161 (3)	0.162 (3)	−0.130 (3)	−0.004 (3)	−0.040 (2)
O10	0.101 (2)	0.179 (3)	0.132 (3)	0.065 (2)	−0.0001 (19)	−0.040 (2)

### Geometric parameters (Å, °)

**Table:**

Cl1—O9	1.372 (3)	C7—H7B	0.9700
Cl1—O10	1.378 (3)	C8—O4	1.423 (3)
Cl1—O8	1.407 (3)	C8—H8A	0.9700
Cl1—O7	1.414 (2)	C8—H8B	0.9700
N1—C13	1.484 (3)	C9—O4	1.421 (3)
N1—H3	0.847 (17)	C9—C10	1.489 (4)
N1—H2	0.836 (18)	C9—H9A	0.9700
N1—H1	0.858 (17)	C9—H9B	0.9700
C1—O6	1.424 (3)	C10—O5	1.416 (3)
C1—C2	1.486 (4)	C10—H10A	0.9700
C1—H1A	0.9700	C10—H10B	0.9700
C1—H1B	0.9700	C11—O5	1.431 (3)
C2—O1	1.427 (3)	C11—C12	1.497 (4)
C2—H2A	0.9700	C11—H11A	0.9700
C2—H2B	0.9700	C11—H11B	0.9700
C3—O1	1.425 (3)	C12—O6	1.425 (3)
C3—C4	1.492 (4)	C12—H12A	0.9700
C3—H3A	0.9700	C12—H12B	0.9700
C3—H3B	0.9700	C13—C14	1.503 (3)
C4—O2	1.417 (3)	C13—H13A	0.9700
C4—H4A	0.9700	C13—H13B	0.9700
C4—H4B	0.9700	C14—C19	1.375 (3)
C5—O2	1.431 (3)	C14—C15	1.380 (4)
C5—C6	1.497 (4)	C15—C16	1.392 (4)
C5—H5A	0.9700	C15—H15A	0.9300
C5—H5B	0.9700	C16—C17	1.360 (4)
C6—O3	1.415 (3)	C16—H16A	0.9300
C6—H6A	0.9700	C17—C18	1.361 (4)
C6—H6B	0.9700	C17—H17A	0.9300
C7—O3	1.421 (3)	C18—C19	1.385 (4)
C7—C8	1.476 (4)	C18—H18A	0.9300
C7—H7A	0.9700	C19—H19A	0.9300
			
O9—Cl1—O10	113.1 (3)	O4—C8—H8B	110.0
O9—Cl1—O8	108.3 (2)	C7—C8—H8B	110.0
O10—Cl1—O8	108.9 (2)	H8A—C8—H8B	108.3
O9—Cl1—O7	109.3 (2)	O4—C9—C10	109.1 (2)
O10—Cl1—O7	108.27 (19)	O4—C9—H9A	109.9
O8—Cl1—O7	108.82 (18)	C10—C9—H9A	109.9
C13—N1—H3	111.3 (18)	O4—C9—H9B	109.9
C13—N1—H2	109 (2)	C10—C9—H9B	109.9
H3—N1—H2	106 (3)	H9A—C9—H9B	108.3
C13—N1—H1	112.5 (19)	O5—C10—C9	108.1 (2)
H3—N1—H1	108 (3)	O5—C10—H10A	110.1
H2—N1—H1	110 (3)	C9—C10—H10A	110.1
O6—C1—C2	110.2 (2)	O5—C10—H10B	110.1
O6—C1—H1A	109.6	C9—C10—H10B	110.1
C2—C1—H1A	109.6	H10A—C10—H10B	108.4
O6—C1—H1B	109.6	O5—C11—C12	113.4 (2)
C2—C1—H1B	109.6	O5—C11—H11A	108.9
H1A—C1—H1B	108.1	C12—C11—H11A	108.9
O1—C2—C1	109.0 (2)	O5—C11—H11B	108.9
O1—C2—H2A	109.9	C12—C11—H11B	108.9
C1—C2—H2A	109.9	H11A—C11—H11B	107.7
O1—C2—H2B	109.9	O6—C12—C11	109.1 (2)
C1—C2—H2B	109.9	O6—C12—H12A	109.9
H2A—C2—H2B	108.3	C11—C12—H12A	109.9
O1—C3—C4	109.2 (2)	O6—C12—H12B	109.9
O1—C3—H3A	109.8	C11—C12—H12B	109.9
C4—C3—H3A	109.8	H12A—C12—H12B	108.3
O1—C3—H3B	109.8	N1—C13—C14	111.97 (19)
C4—C3—H3B	109.8	N1—C13—H13A	109.2
H3A—C3—H3B	108.3	C14—C13—H13A	109.2
O2—C4—C3	109.6 (2)	N1—C13—H13B	109.2
O2—C4—H4A	109.7	C14—C13—H13B	109.2
C3—C4—H4A	109.7	H13A—C13—H13B	107.9
O2—C4—H4B	109.7	C19—C14—C15	118.4 (2)
C3—C4—H4B	109.7	C19—C14—C13	120.2 (2)
H4A—C4—H4B	108.2	C15—C14—C13	121.4 (2)
O2—C5—C6	112.9 (2)	C14—C15—C16	120.6 (2)
O2—C5—H5A	109.0	C14—C15—H15A	119.7
C6—C5—H5A	109.0	C16—C15—H15A	119.7
O2—C5—H5B	109.0	C17—C16—C15	119.9 (3)
C6—C5—H5B	109.0	C17—C16—H16A	120.0
H5A—C5—H5B	107.8	C15—C16—H16A	120.0
O3—C6—C5	108.5 (2)	C16—C17—C18	120.2 (3)
O3—C6—H6A	110.0	C16—C17—H17A	119.9
C5—C6—H6A	110.0	C18—C17—H17A	119.9
O3—C6—H6B	110.0	C17—C18—C19	120.1 (3)
C5—C6—H6B	110.0	C17—C18—H18A	119.9
H6A—C6—H6B	108.4	C19—C18—H18A	119.9
O3—C7—C8	109.8 (2)	C14—C19—C18	120.8 (2)
O3—C7—H7A	109.7	C14—C19—H19A	119.6
C8—C7—H7A	109.7	C18—C19—H19A	119.6
O3—C7—H7B	109.7	C3—O1—C2	112.38 (19)
C8—C7—H7B	109.7	C4—O2—C5	113.7 (2)
H7A—C7—H7B	108.2	C6—O3—C7	112.5 (2)
O4—C8—C7	108.7 (2)	C9—O4—C8	112.3 (2)
O4—C8—H8A	110.0	C10—O5—C11	113.4 (2)
C7—C8—H8A	110.0	C1—O6—C12	111.88 (18)
			
O6—C1—C2—O1	−65.1 (3)	C13—C14—C19—C18	179.5 (2)
O1—C3—C4—O2	68.5 (3)	C17—C18—C19—C14	0.5 (4)
O2—C5—C6—O3	64.3 (3)	C4—C3—O1—C2	178.8 (2)
O3—C7—C8—O4	−66.0 (3)	C1—C2—O1—C3	−176.0 (2)
O4—C9—C10—O5	69.6 (3)	C3—C4—O2—C5	−179.4 (2)
O5—C11—C12—O6	63.4 (3)	C6—C5—O2—C4	84.9 (3)
N1—C13—C14—C19	−93.2 (3)	C5—C6—O3—C7	175.3 (2)
N1—C13—C14—C15	87.6 (3)	C8—C7—O3—C6	176.5 (2)
C19—C14—C15—C16	0.8 (4)	C10—C9—O4—C8	−178.0 (2)
C13—C14—C15—C16	−179.9 (2)	C7—C8—O4—C9	−168.3 (2)
C14—C15—C16—C17	0.4 (4)	C9—C10—O5—C11	178.6 (2)
C15—C16—C17—C18	−1.2 (4)	C12—C11—O5—C10	76.3 (3)
C16—C17—C18—C19	0.8 (4)	C2—C1—O6—C12	−178.3 (2)
C15—C14—C19—C18	−1.3 (4)	C11—C12—O6—C1	177.8 (2)

### Hydrogen-bond geometry (Å, °)

**Table:**

Cg1 is the centroid of the C14–C19 benzene ring.

**Table:**

*D*—H···*A*	*D*—H	H···*A*	*D*···*A*	*D*—H···*A*
N1—H3···O1	0.85 (2)	2.47 (2)	2.955 (3)	117 (2)
N1—H2···O2	0.84 (2)	2.11 (2)	2.938 (3)	174 (3)
N1—H2···O3	0.84 (2)	2.60 (3)	3.003 (3)	111 (2)
N1—H1···O4	0.86 (2)	2.18 (2)	3.004 (3)	161 (3)
N1—H1···O5	0.86 (2)	2.61 (3)	2.968 (3)	107 (2)
N1—H3···O6	0.85 (2)	2.07 (2)	2.885 (3)	162 (3)
C13—H13B···Cg1^i^	0.97	2.99	3.545 (3)	117

Symmetry codes: (i) −*x*+1, −*y*+1, −*z*.

## References

[bb3] Ge, J.-Z., Fu, X.-Q., Hang, T., Ye, Q. & Xiong, R.-G. (2010). *Cryst. Growth Des.* **10**, 3632–3637.

[bb1] Rigaku (2005). *CrystalClear* Rigaku Corporation, Tokyo, Japan.

[bb2] Sheldrick, G. M. (2008). *Acta Cryst.* A**64**, 112–122. 10.1107/S0108767307043930 18156677

